# Brain Sensory Network Activity Underlies Reduced Nociceptive-Initiated and Nociplastic Pain via Acupuncture in Fibromyalgia

**DOI:** 10.21203/rs.3.rs-7086285/v1

**Published:** 2025-10-31

**Authors:** Apeksha Sridhar, Ishtiaq Mawla, Eric Ichesco, Brock Pluimer, Steven Harte, Robert Edwards, Vitaly Napadow, Richard Harris

**Affiliations:** University of California Irvine; University of California Irvine; University of Michigan–Ann Arbor; University of California Irvine; University of Michigan; Perioperative and Pain Medicine, Brigham and Women’s Hospital, Harvard Medical School; Martinos Center for Biomedical Imaging, Massachusetts General Hospital; University of California at Irvine

## Abstract

Chronic pain may arise from overlapping pain pathways, including nociceptive pain driven by peripheral tissue damage and nociplastic pain arising from central nervous system dysregulation, as seen in fibromyalgia. This study investigated how electroacupuncture (EA) produces analgesia by modulating these pain mechanisms via changes in brain activation and functional connectivity (FC). Following 4-weeks of EA, reductions in widespread pain, a marker of central nociplastic pain, were associated with increased pressure-pain tolerance to peripheral nociceptive stimuli. This nociplastic-nociceptive relationship was mediated by increased somatosensory activation (S1) and S1–insula FC, suggesting that EA operates through a “bottom-up” mechanism of action: afferent input from the needle modulates central sensory networks to reduce nociplastic pain. In contrast, the sham group reduced widespread pain via a “top-down” centrally inhibiting mechanism, characterized by decreased precuneus activation and reduced precuneus–insula FC. These findings elucidate contributions of bottom-up versus top-down circuits in mediating pain relief.

## Introduction

Chronic pain conditions are often challenging to treat because of their complex, multifaceted nature and variability among individuals^1^. The current understanding of this complexity is that pain arises from three primary processes: nociceptive, neuropathic, and nociplastic pain mechanisms^2,3,4^. Nociceptive pain arises from tissue damage or inflammation, which activates peripheral nociceptors, whereas neuropathic pain stems from nervous system injury^2,3,4^. In contrast, nociplastic pain is thought to result from central nervous system (CNS) dysfunction, where pain signals are amplified despite the absence of clear peripheral tissue damage, inflammation, nerve lesions or disease^2,3,4^. While these classifications offer a useful framework, many patients with chronic pain display overlapping characteristics of these mechanisms, with multiple pain processes coexisting. Disentangling these characteristics can be crucial for targeted treatments based on nociplastic-dominant versus nociceptive-dominant phenotypes. For instance, chronic pelvic pain often involves both nociceptive components, such as endometriosis or pelvic inflammatory disease, and nociplastic processes characterized by central sensitization. In women with chronic pelvic pain undergoing hysterectomy, those with greater preoperative markers of central sensitization were more likely to experience persistent postoperative pain, highlighting the limited efficacy of peripheral interventions in individuals with primarily nociplastic pain profiles (As-Sanie et al., 2023).

Fibromyalgia (FM) is an example of a chronic pain condition in which both peripherally driven (nociceptive) and central (nociplastic) mechanisms may contribute to chronic pain intensity^5^. FM is primarily characterized by widespread pain but is also associated with fatigue, sleep disturbances, and cognitive impairments^3^. Neuroimaging studies in FM have shown altered brain activity in pain-processing regions, including the insula, anterior cingulate cortex (ACC), and primary somatosensory cortex (S1), in the absence of external stimuli^6^ as well as in response to non-painful and non-somatic sensory input^7,8^, indicating a generalized hypersensitivity in central sensory processing systems. These findings suggest that chronic pain in FM may be perpetuated by abnormal central pain processing, commonly referred to as central sensitization, rather than ongoing peripheral injury^5,9^. Further supporting this, resting-state functional connectivity (FC) studies have shown increased connectivity between the default mode network (DMN) and salience network (SLN), particularly in the anterior insula, which correlates with heightened pain sensitivity and impaired pain modulation^10^. Additionally, altered neurochemistry in the insula has been observed in FM, including reduced levels of γ-aminobutyric acid (GABA)^11^ and elevated levels of glutamate^12^, suggesting impaired inhibitory modulation of pain that contributes to the amplification of widespread pain symptoms in FM.

Individuals with FM also exhibit heightened sensitivity (i.e., reduced pain thresholds) to nociceptive stimuli, such as pressure or heat, suggesting that increased sensitivity to nociceptive pain may also contribute to central nociplastic processes in some patients^13,14^. One of the first studies in FM showed that patients with FM exhibit increased brain activation in pain-processing regions, including the S1 and insula, in response to a peripheral nociceptive stimulus^13^. While it is possible that nociceptive afference may be normal in FM and this is simply amplified in the CNS, another possibility is that a combination of both peripheral nociceptive and central nociplastic mechanisms may both contribute to the complex pain experience in some individuals with FM. Indeed, we have proposed that both peripheral “bottom up” and central “top down” processes may contribute to pain in FM^2^.

Electroacupuncture (EA), a modern variation of traditional acupuncture that involves electrical stimulation delivered via acupuncture needles, has emerged as a promising non-pharmacological treatment for multiple pain disorders, including FM^15^. These clinical findings are supported by neuroimaging studies, which show that EA might target nociplastic pain by modulating activity in pain-processing regions, recalibrating brain connectivity in pain-facilitatory circuits^16–20^, and engaging descending pain inhibitory systems^21^. EA is also shown to enhance the release of endogenous opioids^22^ and modulate neurotransmitters such as endocannabinoids^23^, serotonin, dopamine, and γ-aminobutyric acid (GABA), which are also critical for pain modulation^24,25,26^. In addition to these central effects, EA alleviates nociceptive pain by activating mechanoreceptors in the skin and muscles, which stimulate afferent nerve fibers triggering spinal and supraspinal pain inhibitory pathways, reducing nociceptive input and inflammation^26^.

Although existing findings indicate some central effects of EA, they offer limited insight into the mechanistic pathways by which EA modulates different pain processes. Understanding how EA engages multiple pain mechanisms is essential for tailoring treatments to each patient’s unique pain profile. The lack of a clear framework for matching specific treatment characteristics to individual pain mechanisms likely contributes to variability in outcomes, with interventions succeeding in some patients but failing in others. Our study is one of the first to bridge this critical gap, as we investigate the neural pathways through which EA targets both nociceptive and nociplastic pain mechanisms via somatosensory brain networks in FM. This is an essential first step toward disentangling overlapping pain mechanisms and developing individualized treatments for those with chronic nociplastic pain diagnoses.

## Results

### Increased Pain Tolerance to Nociceptive-Initiated Stimuli Following EA is Associated with Reduced Nociplastic Widespread Pain

To examine changes in nociceptive and nociplastic pain, we examined pressure-pain tolerance (PPTol) and widespread pain scores as PPTol reflects sensitivity to nociceptive pain stimuli, while widespread pain is a clinical marker of nociplastic pain (see Methods). Although neither the EA nor mock laser (ML; sham) group showed statistically significant post-pre changes in pressure-pain tolerance (PPTol) or widespread pain, there was a good deal of inter-individual variability in change scores.

**Figure 1:**
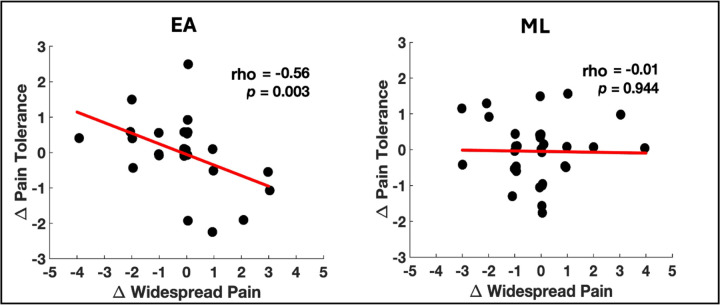
Changes in nociceptive initiated pressure-pain tolerance and nociplastic widespread pain are related in EA but not ML. Scatter plots show the correlation between changes in widespread pain (x-axis; post-pre treatment) and changes in pressure-pain tolerance (y-axis; post-pre treatment in kgf/cm^2^) for the electroacupuncture (EA) group (left) and mock laser (ML) group (right). In the EA group, increased pain tolerance was significantly correlated with reduced widespread pain, while no such relationship was observed in the ML group. Spearman correlation coefficients (rho) and p-values for each group are included in the plots. The red lines indicate the linear regression fits for each group.

In the EA group, increased PPTol was significantly correlated with reduced widespread pain (rho = −0.48, *p* = 0.036), while no such relationship was found in the ML group (rho = 0.14, *p* = 0.501; Fig. 1). In addition, there was a significant group difference in the strength of the correlation between changes in PPTol and widespread pain, with the relationship being significantly stronger in the EA group compared to the ML group (z = −2.0, *p* = 0.04).

### Brain Activation in Sensory and Default Mode Network Regions Mediates the Relationship Between Improved PPTol and Reduced Widespread Pain in the EA Group

**Figure 2:**
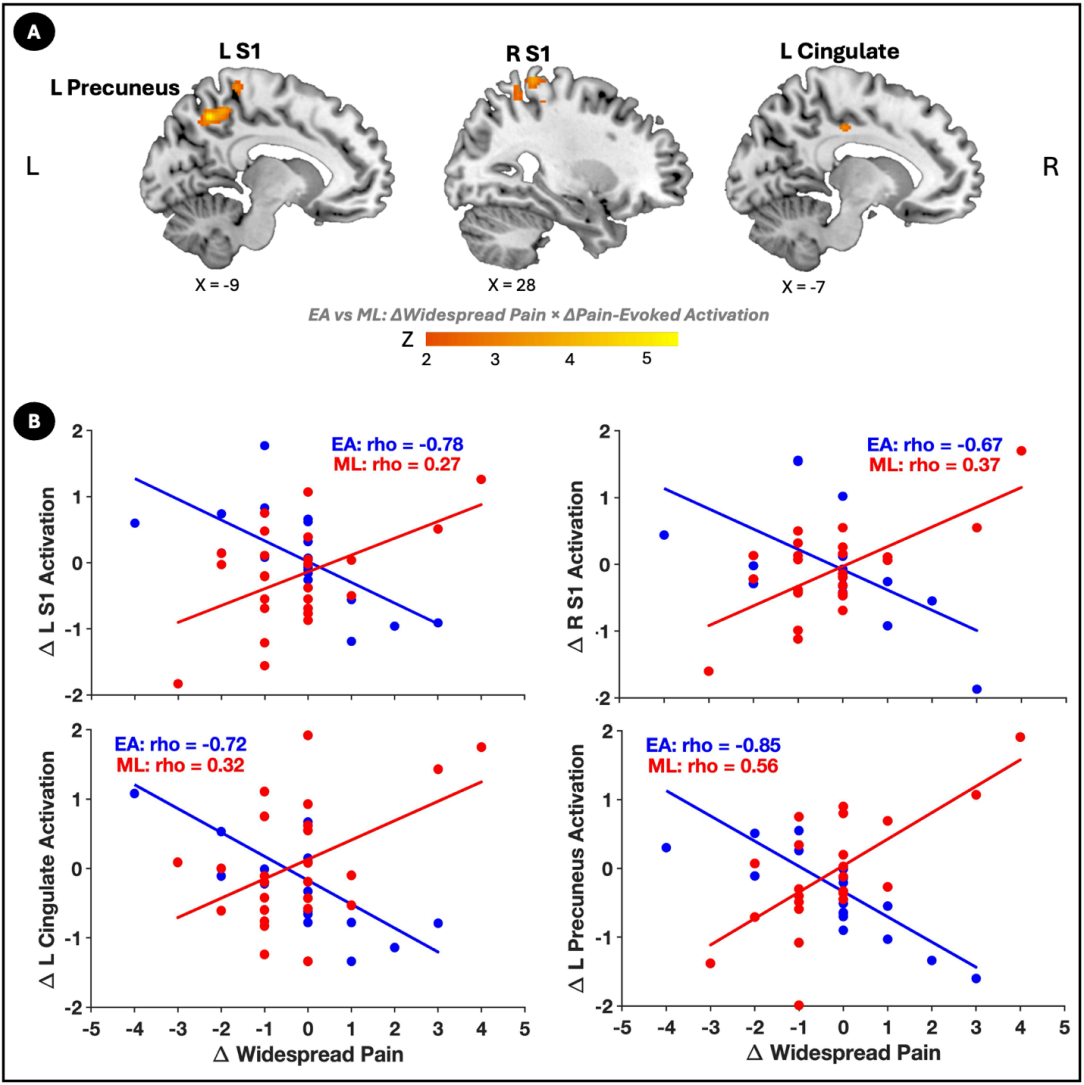
Associations between changes in brain activation and widespread pain differ between EA and ML. (A) Brain regions showing significant differences between EA and ML in how activation changes during evoked pressure-pain relate to changes in widespread pain, including the left and right primary somatosensory cortices (L S1, R S1), left cingulate cortex, and left precuneus. The color bar represents Z-scores. (B) Scatter plots display the correlation between changes in activation (% BOLD signal change, y-axis) and changes in widespread pain (# total body pain regions, x-axis) for the electroacupuncture (EA) group (blue) and mock laser (ML) group (red). In the EA group, greater increases in activation in these regions correlated with larger reductions in widespread pain, while the ML group showed positive correlations, particularly in the left precuneus. Spearman correlation coefficients (rho) and *p*-values are displayed for each group.

Results of a whole-brain activation analysis demonstrated significant group differences in the relationship between changes in brain activations during evoked pressure-pain and changes in widespread pain in four regions: the left and right primary somatosensory cortices (L S1 and R S1), the left cingulate cortex, and the left precuneus (Fig. 2; Table 2). In the EA group, greater activation in all four of these regions was associated with larger reductions in widespread pain. In contrast, the ML group showed positive correlations, particularly in the left precuneus, where reduced activation was linked to decreases in widespread pain (Fig. 2).

Furthermore, in the EA group, activations in three of these regions: right S1 (rho = 0.56, *p* = 0.013), left cingulate cortex (rho = 0.59, *p* = 0.008), and left precuneus (rho = 0.50, *p* = 0.031)—were significantly correlated with improvements in PPTol, which was not observed in the ML group.

The table presents group-level differences between EA and ML where changes in brain activation were differentially correlated with reductions in widespread pain between the two treatment groups (voxel-wise p < 0.001; cluster-wise FWE-corrected p < 0.05). The table includes cluster sizes, peak coordinates, T-statistics, and significance values.

We hypothesized that brain activation would bridge the relationship between improved PPTol and reduced widespread pain, particularly in the EA group. Supporting this, mediation analyses showed that activations in all identified brain regions significantly mediated the link between increased PPTol and reduced widespread pain in the EA group (Table 3; example in Supplemental Fig. 1).

The table presents the direct and indirect effects of changes in brain activation in the left and right primary somatosensory cortices (L S1, R S1), left posterior cingulate cortex (PCC), and left precuneus, individually mediating the relationship between increased pain tolerance (independent variable; X) and reductions in widespread pain (dependent variable; Y). The regression Beta coefficients and bootstrap confidence intervals [lower limit, upper limit] for both the effects are reported for each mediator (M).

### gPPI Analysis Reveal Distinct Connectivity Patterns Linked to Widespread Pain Reduction in EA and ML Groups

**Fig. 4:**
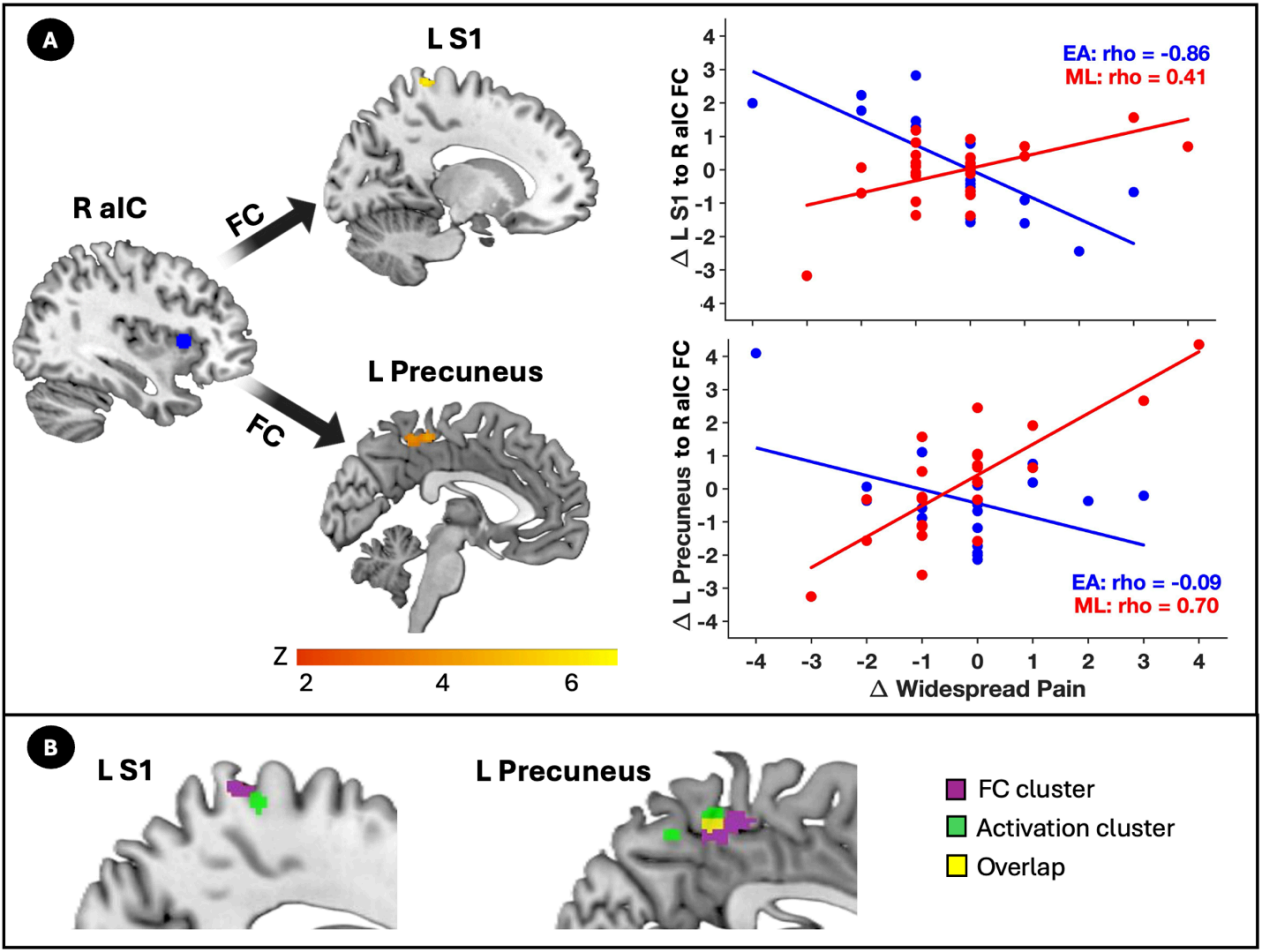
Changes in functional connectivity (FC) during pain and their relationship with widespread pain reduction. (A) Brain regions showing significant differences in the relation of FC changes during evoked pain with widespread pain between the electroacupuncture (EA) group and mock laser (ML) group, including FC between the right anterior insula (R aIC) and left primary somatosensory cortex (L S1), as well as between R aIC and the left precuneus. Scatter plots depict correlations between changes in FC (y-axis) and changes in widespread pain (x-axis) for the EA group (blue) and ML group (red). Spearman correlation coefficients (rho) are reported for each group. (C) Spatial overlap between activation and FC clusters. Although the clusters in the left S1 are non-overlapping, they both fall within the somatotopic hand representation, indicating that they lie in close proximity and may reflect modulation of the same hand-related sensory area by EA. Overlapping activation and FC clusters were observed in the precuneus.

Group comparisons from the gPPI analysis revealed significant differences in the relationship between post-pre change scores in FC during evoked pressure-pain and widespread pain across the EA and ML groups. In the EA group, increases in FC between the right anterior insula seed (R aIC) and left S1 resulting cluster during the pain ramp relative to rest was strongly associated with greater reductions in widespread pain (rho = −0.76, *p* < 0.001; Fig. 4A). A similar association was observed between the left S1 activation cluster seed and a right S1 cluster (rho = −0.84, *p* < 0.001). In contrast, in the ML group, reductions in widespread pain were associated with decreased FC between the R aIC seed and a left precuneus cluster (rho = 0.80, *p* < 0.001; Fig. 4A). Notably, the left S1 clusters identified in the activation and FC analyses were spatially adjacent, while the precuneus clusters showed spatial overlap (Fig. 4B). Detailed information on significant clusters is provided in Supplemental Table 1.

### Modulation of Pain Tolerance and Widespread Pain Through Activation and Subsequent Connectivity in the EA Group

Increased connectivity between left S1 and R aIC was not only correlated with reductions in widespread pain in the EA group but was also positively correlated with activation in left S1. We hypothesized that S1 activation may be contributing to the observed increase in its FC with the anterior insula, which was supported with the mediation analysis showing that FC between left S1 and R aIC mediated the relationship between S1 activation and reductions in widespread pain following treatment (Supplemental Table 2).

A combined serial mediation model was then constructed to examine whether both brain activation and connectivity sequentially mediate the behavioral effects of EA treatment: ΔPPTol (X) → ΔS1 activation (M1) → ΔS1–R aIC connectivity (M2) → Δwidespread pain (Y). The rationale for this model was driven by previous findings^18^, which showed that increased FC between the S1 and R aIC was associated with reductions in clinical pain. Building on this, we sought to delineate the full mechanistic pathway, starting from peripheral nociceptive input, progressing through cortical activation and long-range connectivity changes, to understand how EA-related changes in PPTol might translate into reductions in centrally maintained widespread pain. Importantly, we modeled activation as preceding connectivity as regional activation is thought to initiate and shape subsequent network-level FC, rather than the other way around^28^.

**Fig. 5:**
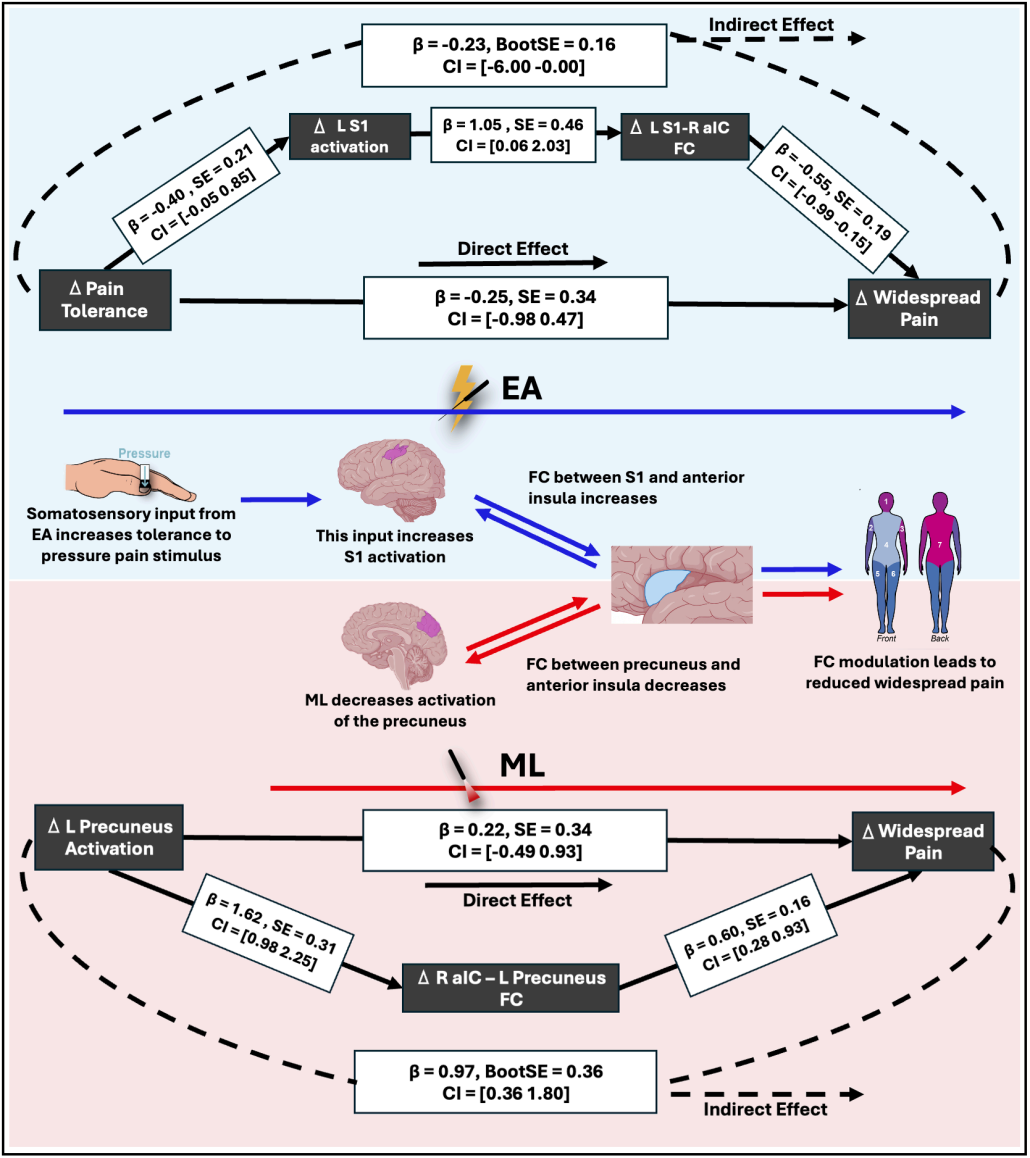
Mediation models for EA and ML examining brain pathways leading to widespread pain reduction. The top model highlighted in blue examines the indirect effects of left primary somatosensory cortex (L S1) activation and L S1 to right anterior insula (R aIC) FC in mediating the relationship between changes in pain tolerance and reductions in widespread pain in the EA group; while the bottom model highlighted in red examines the indirect effects of left precuneus activation and precuneus to R aIC FC in mediating the relationship between changes in pain tolerance and reductions in widespread pain in the EA group. Solid lines represent direct effects, while dashed lines indicate indirect effects. Standardized beta coefficients (β), standard errors (SE), and confidence intervals (CI) are provided for each path. The middle panel illustrates the hypothesized mechanism in the EA (blue) and ML (red) groups.

Both mediators significantly contributed to the pathway, indicating that increased PPTol leads to increased S1 activation, which then facilitates strengthened connectivity to the R aIC, culminating in reduced widespread pain (Fig. 5). Reversing the order of the mediators, placing FC before activation, did not yield significant effects.

In contrast, the ML group exhibited a different mechanism. In this group, reduced FC between the precuneus and the R aIC significantly mediated the relationship between decreased precuneus activation and reduced widespread pain. (Supplemental Table 2).

## Discussion

Our findings highlight the distinction between nociceptive-initiated and nociplastic pain pathways, which often overlap but may require different treatment strategies. Nociceptive pain arises from peripheral injury or stimulation and is transmitted to the central nervous system (CNS) via a “bottom-up” mechanism^2^. In contrast, nociplastic pain may be driven by both “bottom-up” and “top-down mechanisms”, the latter of which highlights dysregulation within the brain and spinal cord^26^.This study examined the mechanistic neural pathway involved in pain reduction during EA treatment for fibromyalgia (FM). Specifically, EA may reduce pain arising from both nociceptive-initiated pressure-pain and widespread nociplastic pain by engaging peripheral sensory input via acupuncture needle afference and then modulating central pain networks.

Our results delineate a bottom-up pathway that begins with acupuncture needle stimulation of the skin and deeper tissues (e.g., muscles, tendons, fascia), activating afferent somatosensory pathways that project to the brain’s primary somatosensory cortex (S1), a region responsible for processing mechanical and tactile input from the body^27^. Such peripheral stimulation has been shown to increase activity in sensory regions such as S1, with greater activation associated with clinical pain reduction, as demonstrated in studies of carpal tunnel syndrome^28^. This activation may then promote stronger long-range connectivity between S1 and the anterior insula (aIC), a region involved in integrating sensory, emotional, and cognitive aspects of pain^29,30^. Within this pathway, S1 activation precedes S1–aIC connectivity, a pattern consistent with prior research indicating that regional activity can initiate and synchronize broader network interactions^31^. These functional changes may, in turn, contribute to a reduction in centrally maintained widespread pain, the hallmark of nociplastic pain in fibromyalgia. Prior work^18^ supports this final stage of the pathway, showing that enhanced S1–aIC connectivity is associated with reductions in pain intensity, an effect mediated by elevated GABA levels in the insula. This suggests that shifts in neurotransmitter signaling within pain-modulatory regions may support the FC changes necessary for analgesia.

Together, these results indicate that EA may engage a sequential “bottom-up” mechanism: somatosensory input first influences local cortical activity in S1, which in turn modulates cortico-cortical FC with regions like the aIC, ultimately leading to reductions in widespread pain. This pathway, from peripheral stimulation to cortical activation, and from cortical activation to network-level integration, may be the mechanism through which EA exerts its analgesic effects. While these findings highlight a bottom-up pathway, they do not preclude the contribution of central influences, such as expectancy, contextual cues, or patient–practitioner interaction, that may also shape EA’s therapeutic effects^32^.

In contrast, ML, a sham intervention devoid of somatosensory signaling, likely operates more through a “top-down” mechanism on widespread nociplastic pain by targeting the precuneus, a key node of the default mode network (DMN) involved in introspection and self-referential thought, which are processes that may contribute to negative and maladaptive cognitive patterns in individuals suffering from chronic pain^33^. Hyperactivity in the DMN has been consistently linked to heightened pain perception and emotional distress in chronic pain conditions, suggesting that deactivation of the DMN and reduction of DMN-insula connectivity may be potential mechanisms for attenuating widespread pain^34–37^. By reducing precuneus activation and its connectivity to the right anterior insula, ML may disrupt these maladaptive cognitive and emotional patterns, thereby contributing to pain relief. This finding aligns with prior research on placebo analgesia, which has similarly demonstrated changes in DMN activity and connectivity that modulate the cognitive-affective aspects of pain through expectation and belief^38,39^. Similar effects were observed in our recent study of cognitive behavioral therapy (CBT), a top-down therapy, for chronic low back pain, where reductions in DMN–insula connectivity were strongly associated with pain relief^40^. Together, these findings suggest that DMN–insula decoupling may represent a shared mechanism underlying top-down analgesic effects across both psychological and expectation-driven interventions.

A major conceptual limitation of this study lies in the absence of gold-standard assays or experimental design to clearly distinguish between nociceptive and nociplastic pain mechanisms. While we interpret increased pressure-pain tolerance as a reflection of nociceptive-initiated pain, and widespread pain as a marker of nociplastic pain, these measures are not entirely distinct. Evoked pressure-pain tolerance, though peripherally driven, is also influenced by central modulation, and widespread pain, which is typically associated with central sensitization, may still be partially sustained by ongoing peripheral nociceptive input. This overlap complicates the attribution of specific neural changes to a single pain mechanism. Nevertheless, based on current scientific understanding and the tools available, these measures represent the best available proxies for approximating nociceptive and nociplastic pain dimensions. In future studies, techniques such as microneurographic recordings from primary afferent fibers may help to more robustly segregate peripherally-driven from centrally-driven effects.

While the reduction in widespread pain did not reach statistical significance in the current sample, prior studies have shown that EA can significantly reduce widespread pain^41^. Similarly, prior work^18^ from our group demonstrated that EA significantly reduced pain severity compared to ML, as measured by the BPI, supporting the clinical relevance of EA. Building on these findings, the present study provides new insights into the brain mechanisms underlying these clinical effects, showing how changes in pressure-pain tolerance and widespread pain are linked through specific brain circuits involved in sensory processing and integration. Future research should focus on identifying and validating moderators to refine patient selection and optimize therapeutic strategies. Studies should also investigate the applicability of these findings to other chronic pain conditions that share overlapping pain mechanisms such as chronic low back pain, pelvic pain, and migraine.

Patients with FM exhibit significant variability in how much each of these pain mechanisms contribute to their symptoms. Some patients may experience predominantly nociplastic pain due to CNS dysfunction, whereas others may have a larger nociceptive component driven by peripheral abnormalities. This variability highlights the importance of tailoring treatment approaches to the type of pain mechanisms in each patient. Treatments such as EA may be particularly beneficial for patients with mixed pain profiles, as they address both nociceptive and nociplastic components, whereas more “top-down” centrally acting treatments such as CBT may be more effective for patients with less nociceptive initiated pain. Although this description focuses on nociceptive and nociplastic mechanisms for clarity, other pain types, such as neuropathic pain, may also play a role and should be considered in a comprehensive, individualized treatment strategy. Understanding the balance between these mechanisms in each patient is crucial for optimizing treatment outcomes and advancing personalized pain management strategies for pain disorders.

## Methods

### Overall Protocol

This study was a single-site, blinded, sham-controlled, randomized, non-crossover longitudinal neuroimaging trial. It was pre-registered with ClinicalTrials.gov (NCT02064296 ) and conducted at the University of Michigan, Ann Arbor, MI, between December 2014 and November 2019. The study received approval from the University of Michigan Medical Institutional Review Board, and all participants provided written informed consent in line with the Declaration of Helsinki.

Some neuroimaging findings from this dataset have been previously published^18,19,42^. However, this study newly reports relationships between brain activity and connectivity to evoked pain and resulting changes in nociceptive-initiated and nociplastic pain following EA.

### Participants

Participants diagnosed with FM were recruited for this study and met the 2011 FM Survey Criteria, self-reported symptoms for at least one year, pain on more than 50% of days, and a pain score of ≥ 4 on a 10 cm Visual Analog Scale (VAS). Exclusion criteria included having received acupuncture within the last 6 months, neurological disorders such as peripheral neuropathy, psychiatric conditions (e.g., schizophrenia, major depression with suicidal ideation), substance abuse, use of opioids or stimulant medications, and contraindications to MRI or electrostimulation. Further details on inclusion/exclusion criteria and medication usage are available in our previous publication^18^.

Participants were randomized into EA or mock laser (ML), a sham control mimicking acupuncture without somatosensory afference (described below). Of the 70 participants with clinical data, 26 were excluded due to missing either baseline or post-treatment evoked pain scans (Supplemental Fig. 1). No participants were excluded for excessive motion (see details below), resulting in a final fMRI sample of 44 participants (EA: 19, ML: 25). All participants completed behavioral assessments, quantitative sensory testing (QST), as well as neuroimaging sessions at the University of Michigan. Clinical characteristics and participant demographics for both the full sample and the fMRI subsample are provided in Supplemental Tables 3 and 4.

### Experimental Design

**Fig. 6:**
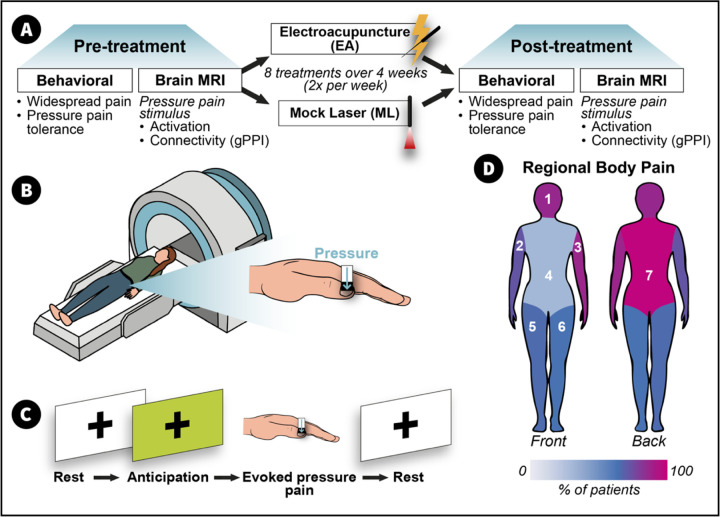
Experimental Design and Pain Assessments. (A) Timeline of the study, including baseline assessments (left), treatment with electroacupuncture (EA) or control mock laser (ML) acupuncture (center), and post-treatment evaluations (right). (B) Illustration of the nociceptive initiated pressure-pain stimulus applied during fMRI sessions. (C) Block design used in fMRI scans, showing rest, anticipation, and evoked pressure-pain phases. (D) Seven body regions used to assess widespread nociplastic pain, with colors representing the percentage of participants reporting pain in each region for both groups combined prior to treatment.

A full description of our study design is displayed in Fig. 6. In brief, participants first underwent a screening session to confirm eligibility and familiarize themselves with the study procedures. They were then randomized using a computer-generated randomization sequence to receive either EA or ML. The comparison between EA and ML was designed to isolate the effects of somatosensory stimulation: EA involves direct sensory input via needle insertion and electrical stimulation, whereas ML serves as a sensory-inactive control, enabling the examination of brain and behavioral responses specifically driven by afferent somatosensory signaling. Behavioral and MRI data were collected from participants before and after treatment (Fig. 6A).

QST included an assessment of pressure-pain tolerance (PPTol) during which pressure stimuli were applied to the left thumbnail bed using the Multimodal Ascending Sensory Test (MAST) system (Arbor Medical Innovations, Saline, MI)^42,43^. A series of discrete pressure stimuli were delivered in ascending order, starting at 0.25 kgf/cm^2^ and increasing in steps of 0.25–0.50 kgf/cm^2^ to a maximum of 10.0 kgf/cm^2^. Participants rated the magnitude of pain evoked at each pressure level on a digital 0–100 Numerical Rating Scale (NRS), anchored at 0 (“no pain”) and 100 (“worst pain imaginable”). Testing stopped when the maximum pressure was reached, or when participants provided a rating of ≥ 80/100 or indicated they were unwilling to continue testing. PPTol was defined as the last pressure recorded during testing before stopping. In addition, stimulus-response data from the entire ascending series were used to interpolate individualized P30 thresholds for each participant, defined as an approximation of the pressure intensity that evokes a rating of 30 on the NRS. P30 thresholds were used in subsequent magnetic resonance imaging (MRI) sessions.

The spatial distribution of pain across the body (widespread pain) was assessed using the FM Survey Criteria^44^. The 19 body sites from the FM Survey Criteria’s Widespread Pain Index subscale were grouped into seven regions: head, front, back, right arm, right leg, left arm, and left leg (Fig. 6D). This consolidation of pain locations into seven primary regions aimed to provide a more accurate representation of widespreadness. Changes in widespread pain were quantified by calculating the difference in the total number of body regions endorsed as painful prior to and following treatment (i.e., post-treatment values minus pre-treatment values).

To assess treatment-related changes in PPTol and widespread pain, we conducted paired-samples t-tests using SPSS (IBM Statistical Package for the Social Sciences, version 29.0.2.0) to determine whether each measure showed a significant change from pre- to post-treatment (at *p* < 0.05). To examine the relationship between PPTol and widespread pain, we performed Spearman correlation analyses in SPSS. In addition, Cocor was used to statistically compare the strength of correlations between groups.

Following each behavioral session, participants completed a functional MRI session that included a block-design task involving alternating periods of rest, pressure pain applied to the left thumbnail bed, and a green cross visually displayed on a monitor indicating the anticipation of pressure stimulation (Fig.s 6B and 6C). Pressure stimuli during the pain blocks were delivered in the scanner using an MRI-compatible device (IPC-1000 Thumb Stimulator; Arbor Medical Innovations, Saline, MI), which applied calibrated pressure through a handpiece connected via tubing routed through the MRI access port. Stimulus timing was precisely synchronized with the experimental protocol using E-Prime software. During the entire scan participants were instructed to remain still with their eyes open, and to focus on the screen. Pain ratings were recorded after each stimulation block on a 0–100 NRS scale. Each block consisted of six 10-second P30 pressure pulses, separated by a 21.5-second inter-stimulus interval (ISI). In addition to the block-design task, a resting-state scan (at the beginning of the scan session), proton magnetic resonance spectroscopy (^1^H-MRS) and other experimental scans were collected, including tests involving pressure on the calf; however, these scans were not included as they have been reported on previously^18,19^.

### Acupuncture Treatment

Participants in the EA and ML groups underwent eight treatment sessions, administered twice weekly over four weeks. In the EA group, participants received electrical stimulation at three pairs of acupoints: right LI-11 to LI-4, left GB-34 to SP-6, and bilateral ST-36. These acupoints were selected based on their clinical relevance to common FM symptoms, such as multisite pain, headaches, gastrointestinal dysfunction, disrupted sleep, and chronic fatigue^45^. A low-frequency EA device delivered electrical stimulation with pulse width, frequency, and intensity individualized to each participant, based on their sensory and pain thresholds. The electrostimulation targeted central pain pathways thought to modulate widespread pain. Sessions lasted 25 minutes. Additional details about the treatment protocol, including the specific parameters of electrostimulation, are provided previously^18^.

In the ML sham control group, participants were told they would receive acupoint stimulation using a laser device; however, the device was inactive and emitted no therapeutic laser energy. This intervention mimicked the appearance and procedure of real treatment while lacking any somatosensory stimulation, serving as a sham control for all EA analyses. This authorized deception was approved by the University of Michigan IRB. The ML device was positioned over the same acupoints as in the EA group. To ensure blinding and credibility, both groups wore blindfolds during treatment and received identical verbal instructions and treatment durations. Participants were fully debriefed after the study. Additionally, a Credibility Questionnaire was administered after the first and last treatment sessions to evaluate participants’ perceptions of the treatment’s validity, ensuring that any observed clinical or neuroimaging differences were not influenced by disparities in treatment credibility. Treatment credibility was found to be equal across both groups.

### MRI Acquisition and Preprocessing

Neuroimaging was conducted using a 3.0T Philips Ingenia MRI scanner at the University of Michigan. High-resolution anatomical images were acquired using a T1-weighted sequence (repetition time [TR] = 8.2 ms, echo time [TE] = 3.7 ms, matrix = 240 × 240, flip angle = 8°, field of view (FOV) = 256 mm, voxel size = 1 mm^3^, 172 slices), providing detailed structural information for subsequent coregistration with functional data. Functional MRI (fMRI) data were collected using a T2*-weighted echo-planar sequence to measure blood oxygenation level-dependent (BOLD) signals during task-based conditions. Each task fMRI scan had the following parameters: volumes = 102, TR = 2 seconds, TE = 30 ms, flip angle = 90°, image size = 2.75 × 2.75 mm^2^, matrix = 80 × 80, slice number = 38, and slice thickness = 3.5mm.

fMRI data were preprocessed using fMRIPrep^46^ (v23.2.0), which included skull stripping, motion correction via rigid body realignment, and nonlinear spatial normalization to MNI152 standard space. Functional images were smoothed with a 6 mm FWHM Gaussian kernel using AFNI’s 3dBlurInMask to enhance signal-to-noise ratio^47^. Framewise displacement (FD) was calculated for each run, and volumes exceeding 0.5 mm FD were flagged for motion scrubbing^48,49^. Participants were excluded from analysis if more than 30% of volumes in a run exceeded the 0.5 mm FD threshold^50^; however, no participants in the present dataset met this exclusion criterion. Additionally, nuisance regressors, including six head motion parameters (translation and rotation) and CompCor components, were included to further denoise the data and remove physiological noise from the functional connectivity (FC) analysis.

### Activation Analysis

First-level statistical analyses were conducted using Statistical Parametric Mapping (SPM12). A general linear model (GLM) was applied to model BOLD responses during the pressure-pain task conditions. The model included four conditions: rest (10–20 seconds), green fixation cross (anticipatory, no painful stimulation; 4–10 seconds), pressure-pain ramp (4 seconds), and pressure-pain plateau (6 seconds). The pain ramp condition specifically targeted the initial 4 seconds of the pain condition to capture early pain perception. This approach was based on evidence that modeling the onset (on-ramp) and offset (off-ramp) periods of pain provides greater sensitivity in detecting brain responses than modeling the full pain block duration^51^. Condition onsets and durations for each condition were modeled according to the experimental design. A high-pass filter with a 128-second cutoff was applied to remove low-frequency drifts in the data. To account for head motion, as mentioned above, confound regressors were generated using FD values, with regressors censoring high-motion time points. The primary contrast of interest, pain ramp > rest, was used to assess brain activity during the early phase of pain perception.

To evaluate intervention-related changes in brain activation, subject-level difference maps were created in SPM using spm_imcalc, subtracting pre-intervention contrast images from post-intervention images. These maps were entered into a group-level analysis using a flexible factorial design in SPM12, with Group (EA vs. ML) as a factor. Age (centered at the overall mean) was included as a covariate of no interest, and changes in widespread pain was included as a covariate of interest. Clusters of activation were identified based on group differences in the relationship between changes in widespread pain and brain activation during the pressure-pain ramp versus rest condition. To focus the analysis on brain tissue, threshold masking was applied using the FSL MNI152 template to exclude non-brain regions. Voxel-wise inference was performed at p < 0.001 (uncorrected), with cluster-level family-wise error (FWE) correction at p < 0.05 to adjust for multiple comparisons.

### Generalized Psychophysiological Interaction (gPPI) Analysis

A Generalized Psychophysiological Interaction (gPPI) analysis was conducted using the CONN toolbox (v.20.b) to examine task modulated FC during pressure-pain stimulation. This approach was chosen because gPPI allows for the examination of task-dependent changes in connectivity across multiple conditions, providing a more comprehensive understanding of context-specific neural interactions compared to traditional correlation-based FC measures^52^. Six regions of interest (ROIs) within the insula were selected as seeds: the left and right anterior, middle, and posterior insula^53^ (radius = 6mm) due to their established role in pain processing and their known aberrant FC in FM patients, which has been linked to altered pain perception^54^. Additionally, activation clusters in the primary somatosensory cortex, anterior cingulate, and precuneus, resulting from the activation analysis were included as seeds to capture task-specific brain regions involved in pain processing (Table 1).

Each region of interest (ROI) in the table is identified by its anatomical label and hemisphere (L = left, R = right), along with the number of voxels included in the ROI mask and its Montreal Neurological Institute (MNI) coordinates (x, y, z). Insular subregions are labeled as anterior (aIC), middle (mIC), or posterior (pIC). S1 = primary somatosensory cortex.

Similar to the activation analysis, four conditions were modeled for both pre- and post-treatment sessions: rest, green fixation cross, pain ramp, and pain plateau. The primary contrast of interest was (pain ramp > rest) post-treatment minus (pain ramp > rest) pre-treatment, designed to assess related treatment-changes in FC during the pain ramp condition. In the second-level analysis, changes in widespread pain were included as a covariate to examine how FC changes related to reductions in pain regions and whether these effects differed between the EA and ML groups. Statistical analyses were performed using a voxel-wise threshold of *p* < 0.001 (uncorrected), with cluster-level family-wise error (FWE) correction at *p* < 0.05 to adjust for multiple comparisons.

### Mediation Analyses

Mediation analyses were conducted to investigate the neural mechanisms linking changes in PPTol and widespread pain in the EA group. Specifically, we examined whether brain activation and FC measures from the gPPI analysis served as mediators in this relationship.

Two models were tested using SPSS’s PROCESS macro^55^ to evaluate mediation and serial mediation pathways. In the first model (Model 4), we tested a simple mediation framework where the independent variable (IV) was the change in PPTol following treatment, the dependent variable (DV) was the change in widespread pain, and the mediator was brain activation in pain-processing regions. This model assessed whether the relationship between improved nociceptive pain tolerance and reduced nociplastic pain was explained by activation changes in activated brain areas.

The second model (Model 6) applied a serial mediation framework incorporating two mediators in sequence: brain activation (M1) and FC between the activation cluster and other brain regions (M2). This model tested a theoretical pathway in which increased somatosensory input from EA leads to heightened primary somatosensory cortex (S1) activation (M1), which then enhances its connectivity with regions like the anterior insula (M2), ultimately resulting in a reduction in widespread pain.

All mediation analyses were conducted using bias-corrected bootstrapping with 5,000 bootstrap samples to estimate indirect effects. Statistical significance was assessed with 95% confidence intervals, with indirect effects considered statistically significant if the confidence interval did not include zero. All mediation analyses were controlled for age.

## Supplementary Files

This is a list of supplementary files associated with this preprint. Click to download.

- CONSORTflowdiagram.doc
- AcuASupplemental.docx
- Protocol.docx

## Figures and Tables

**Table 1:** Regions of interest (ROIs) used for gPPI analysis

Seed ROI	Number of Voxels (k)	MNI Coordinates (x,y,z)
Left anterior insula (L aIC)	123	−32, 16, 6
Left middle insula (L mIC)	123	−38, 2, 8
Left posterior insula (L pIC)	136	−39, −15, 11
Right anterior insula (R aIC)	123	32, 16, 6
Right middle insula (R mIC)	123	38, 2, 8
Right posterior insula (R pIC)	112	39, −15, 8
Left somatosensory cortex (L S1)	75	−8, −38, 68
Right somatosensory cortex (R S1)	438	20, −34, 66
Left cingulate cortex	17	−14, −26, 38
Left Precuneus	223	−8, −58, 48

**Table 2:** Group-level differences between EA and ML treatments in associations between changes in brain activation and widespread pain.

Cluster	Cluster Size (k)	ML > EA (T-statistic, *p*-value)	Peak Coordinates (x, y, z)
L S1	75	T = 4.32, *p* = 0.005	−8, −38, 68
R S1	438	T = 4.66, *p* < 0.001	20, −34, 66
L cingulate	17	T = 4.63, *p* < 0.001	−14, −26, 38
L precuneus	223	T = 4.67, p = 0.001	−8, −58, 48

**Table 3:** Brain Activations Mediate the Effects of Increased Pressure-pain Tolerance on Reductions in Body Pain Regions Following EA

Path		Mediator (M)			
		L S1	R S1	L PCC	L Precuneus
Path a: Pain Tolerance (X) → Brain Activation (M)	Beta (SE)	0.41 (0.23)	0.37 (0.25)	0.62 (0.24)	0.43 (0.26)
	CI	[−0.05, 0.85]	[−0.12, 0.87]	[0.13, 1.10]	[−0.07, 0.94]
Path b: Brain Activation (M) → Widespread Pain (Y)	Beta (SE)	−1.25 (0.45)	−0.98 (0.43)	−0.99 (0.45)	−1.56 (0.38)
	CI	[−2.14, −0.35]	[−1.84, −0.13]	[−1.87, −0.11]	[−1.94, −0.40]
Path c: Pain Tolerance (X) → Widespread Pain (Y)	Beta (SE)	−0.42 (0.43)	−0.55 (0.45)	−0.31 (0.50)	−0.41 (0.42)
	CI	[−1.28, 0.44]	[−1.44, 0.34]	[−1.30, 0.69]	[−1.24, 0.41]
Path c′: Pain Tolerance (X) → Widespread Pain (Y) after controlling for brain activation (M)	Beta (SE)	−0.50 (0.27)	−0.37 (0.29)	−0.61 (0.38)	−0.50 (0.34)
	CI	[−1.16, −0.09]	[−1.22, −0.04]	[−1.76, −0.20]	[−1.59, −0.12]

## Data Availability

The datasets generated and/or analyzed in the current study are available from the corresponding author upon request.

## References

[1] Fornasari D (2012) Pain mechanisms in patients with chronic pain. Clin Drug Investig 32(Suppl 1):45–52 10.2165/11630070-000000000-00000 23389875

[2] Kaplan CM (2024) Deciphering nociplastic pain: clinical features, risk factors and potential mechanisms. Nat Rev Neurol 20:347–363 38755449 10.1038/s41582-024-00966-8

[3] Clauw DJ (2014) Fibromyalgia: a clinical review. JAMA 311:1547–1555 24737367 10.1001/jama.2014.3266

[4] Fitzcharles M-A, Arnold LM, Häuser W, Clauw DJ (2024) Nociplastic pain: towards an understanding of prevalent pain conditions. Nat Rev Neurol 20:261–275

[5] Sluka KA, Clauw DJ (2016) Neurobiology of fibromyalgia and chronic widespread pain. Neuroscience 338:114–129 27291641 10.1016/j.neuroscience.2016.06.006 PMC5083139

[6] Dehghan M (2016) Coordinate-based (ALE) meta-analysis of brain activation in patients with fibromyalgia. Hum Brain Mapp 37:1749–1758 26864780 10.1002/hbm.23132 PMC6867581

[7] Harte SE (2014) Pharmacologic attenuation of cross-modal sensory augmentation within the chronic pain insula. Pain 155:943–950 10.1097/j.pain.0000000000000593 PMC4988086 27101425

[8] López-Solà M (2014) Altered functional magnetic resonance imaging responses to nonpainful sensory stimulation in fibromyalgia patients. Arthritis Rheumatol 66:3200–3209 25220783 10.1002/art.38781 PMC4410766

[9] Harte SE, Harris RE, Clauw DJ (2018) The neurobiology of central sensitization. J Appl Biobehav Res 23:e12137

[10] Napadow V (2010) Intrinsic brain connectivity in fibromyalgia is associated with chronic pain intensity. Arthritis Rheum 62:2545–2555 20506181 10.1002/art.27497 PMC2921024

[11] Foerster BR (2012) Reduced insular γ-aminobutyric acid in fibromyalgia. Arthritis Rheum 64:579–583 21913179 10.1002/art.33339 PMC3374930

[12] Harris RE (2009) Elevated insular glutamate in fibromyalgia is associated with experimental pain. Arthritis Rheum 60:3146–3152 19790053 10.1002/art.24849 PMC2827610

[13] Gracely RH, Petzke F, Wolf JM, Clauw DJ (2002) Functional magnetic resonance imaging evidence of augmented pain processing in fibromyalgia. Arthritis Rheum 46:1333–1343 12115241 10.1002/art.10225

[14] Staud R, Vierck CJ, Cannon RL, Mauderli AP, Price DD (2001) Abnormal sensitization and temporal summation of second pain (wind-up) in patients with fibromyalgia syndrome. Pain 91:165–175 11240089 10.1016/s0304-3959(00)00432-2

[15] Deare JC (2013) Acupuncture for treating fibromyalgia. Cochrane Database Syst. Rev. CD007070 (2013) 23728665 10.1002/14651858.CD007070.pub2 PMC4105202

[16] Rodrigues JM (2024) Electro-acupuncture effects measured by functional magnetic resonance imaging—A systematic review of randomized clinical trials. Healthcare 12:2 10.3390/healthcare12010002 PMC10778902 38200908

[17] Ha G (2022) Coordinate-based (ALE) meta-analysis of acupuncture for musculoskeletal pain. Front Neurosci 16:906875 35937886 10.3389/fnins.2022.906875 PMC9354890

[18] Mawla I (2021) Greater somatosensory afference with acupuncture increases primary somatosensory connectivity and alleviates fibromyalgia pain via insular γ-aminobutyric acid: a randomized neuroimaging trial. Arthritis Rheumatol 73:1318–1328 33314799 10.1002/art.41620 PMC8197768

[19] Mawla I (2023) Large-scale momentary brain co-activation patterns are associated with hyperalgesia and mediate focal neurochemistry and cross-network functional connectivity in fibromyalgia. Pain 164:2737–2748 37751539 10.1097/j.pain.0000000000002973 PMC10652715

[20] Napadow V, Kim J, Clauw DJ, Harris RE (2012) Decreased intrinsic brain connectivity is associated with reduced clinical pain in fibromyalgia. Arthritis Rheum 64:2398–2403 22294427 10.1002/art.34412 PMC3349799

[21] Lv Q (2022) The involvement of descending pain inhibitory system in electroacupuncture-induced analgesia. Neural Plast. 6262463 (2022) 10.3389/fnint.2019.00038 PMC6712431 31496944

[22] Wang Y (2014) CXCL10 controls inflammatory pain via opioid peptide-containing macrophages in electroacupuncture. PLoS Biol 12:e1001755 10.1371/journal.pone.0094696 PMC3986408 24732949

[23] Ho T-J (2021) Electroacupuncture attenuates inflammatory pain via peripheral cannabinoid receptor type 1 signalling pathways in mice. Sci Rep 11:1295 38060514 10.1371/journal.pone.0295432 PMC10703209

[24] Han JS (2004) Acupuncture and endorphins. Neurosci Lett 361:258–261 15135942 10.1016/j.neulet.2003.12.019

[25] Zhao ZQ (2008) Neural mechanism underlying acupuncture analgesia. Prog Neurobiol 85:355–375 18582529 10.1016/j.pneurobio.2008.05.004

[26] Zhang R, Lao L, Ren K, Berman BM (2014) Mechanisms of acupuncture-electroacupuncture on persistent pain. Anesthesiology 120:482–503 24322588 10.1097/ALN.0000000000000101 PMC3947586

[27] Ziegler K (2023) Primary somatosensory cortex bidirectionally modulates sensory gain and nociceptive behavior in a layer-specific manner. Nat Commun 14:2999 37225702 10.1038/s41467-023-38798-7 PMC10209111

[28] Liu X, Zhang N, Chang C, Duyn JH (2018) Co-activation patterns in resting-state fMRI signals. NeuroImage 180, 485–494 29355767 10.1016/j.neuroimage.2018.01.041 PMC6082734

[29] Maeda Y (2013) Acupuncture-evoked response in somatosensory and prefrontal cortices predicts immediate pain reduction in carpal tunnel syndrome. Evid. Based Complement. Alternat. Med. 795906 (2013) 23843881 10.1155/2013/795906 PMC3703406

[30] Menon V (2025) Insular cortex: a hub for saliency, cognitive control, and interoceptive awareness. Encyclopedia Hum Brain 159–183

[31] Kim JH (2017) Impaired insula functional connectivity associated with persistent pain perception in patients with complex regional pain syndrome. PLoS ONE 12:e0180479 28692702 10.1371/journal.pone.0180479 PMC5503260

[32] Necka EA (2019) Applications of dynamic functional connectivity to pain and its modulation. Pain Rep 4:e752 31579848 10.1097/PR9.0000000000000752 PMC6728009

[33] Ellingsen DM (2020) Dynamic brain-to-brain concordance and behavioral mirroring as a mechanism of the patient-clinician interaction. Sci Adv 6:eabc1304 33087365 10.1126/sciadv.abc1304 PMC7577722

[34] Dadario NB, Sughrue ME (2023) The functional role of the precuneus. Brain 146:3598–3607 37254740 10.1093/brain/awad181

[35] van Ettinger-Veenstra H (2019) Chronic widespread pain patients show disrupted cortical connectivity in default mode and salience networks, modulated by pain sensitivity. J Pain Res 12:1743–1755 31213886 10.2147/JPR.S189443 PMC6549756

[36] Baliki MN, Geha PY, Apkarian AV, Chialvo DR (2008) Beyond feeling: chronic pain hurts the brain, disrupting the default-mode network dynamics. J Neurosci 28:1398–1403 18256259 10.1523/JNEUROSCI.4123-07.2008 PMC6671589

[37] Cottam WJ, Iwabuchi SJ, Drabek MM, Reckziegel D, Auer DP (2018) Altered connectivity of the right anterior insula drives the pain connectome changes in chronic knee osteoarthritis. Pain 159:929–938 29557928 10.1097/j.pain.0000000000001209 PMC5916486

[38] Wager TD (2004) Placebo-induced changes in FMRI in the anticipation and experience of pain. Science 303:1162–1167 14976306 10.1126/science.1093065

[39] Petrovic P, Kalso E, Petersson KM, Ingvar M (2002) Placebo and opioid analgesia—imaging a shared neuronal network. Science 295:1737–1740 11834781 10.1126/science.1067176

[40] Lee J (2024) A randomized controlled neuroimaging trial of cognitive behavioral therapy for fibromyalgia pain. Arthritis Rheumatol 76:130–140 37727908 10.1002/art.42672 PMC10842345

[41] Deluze C, Bosia L, Zirbs A, Chantraine A, Vischer TL (1992) Electroacupuncture in fibromyalgia: results of a controlled trial. BMJ 305:1249–1252 1477566 10.1136/bmj.305.6864.1249 PMC1883744

[42] Murphy AE (2024) Temporal summation but not expectations of pain relief predict response to acupuncture treatment in fibromyalgia. J Pain 25:104622 38986891 10.1016/j.jpain.2024.104622

[43] Harte SE (2013) Development and validation of a pressure-type automated quantitative sensory testing system for point-of-care pain assessment. Med Biol Eng Comput 51:633–644 23381890 10.1007/s11517-013-1033-x

[44] Wolfe F (2011) Fibromyalgia criteria and severity scales for clinical and epidemiological studies: a modification of the ACR Preliminary Diagnostic Criteria for Fibromyalgia. J Rheumatol 38:1113–1122 21285161 10.3899/jrheum.100594

[45] Harris RE (2005) Treatment of fibromyalgia with formula acupuncture: investigation of needle placement, needle stimulation, and treatment frequency. J Altern Complement Med 11:663–671 16131290 10.1089/acm.2005.11.663

[46] Esteban O (2019) fMRIPrep: a robust preprocessing pipeline for functional MRI. Nat Methods 16:111–116 30532080 10.1038/s41592-018-0235-4 PMC6319393

[47] Cox RW (1996) AFNI: software for analysis and visualization of functional magnetic resonance neuroimages. Comput Biomed Res 29:162–173 8812068 10.1006/cbmr.1996.0014

[48] Power JD (2012) Spurious but systematic correlations in functional connectivity MRI networks arise from subject motion. NeuroImage 59:2142–2154 22019881 10.1016/j.neuroimage.2011.10.018 PMC3254728

[49] Power JD (2014) Methods to detect, characterize, and remove motion artifact in resting state fMRI. NeuroImage 84:320–341 23994314 10.1016/j.neuroimage.2013.08.048 PMC3849338

[50] Smith J (2022) Can this data be saved? Techniques for high motion in resting state scans of first grade children. Dev Cogn Neurosci 58:101178 36434964 10.1016/j.dcn.2022.101178 PMC9694086

[51] Smith JK, Humes DJ, Head KE (2011) fMRI and MEG analysis of visceral pain in healthy volunteers. Neurogastroenterol Motil 23:648–e260 21507149 10.1111/j.1365-2982.2011.01712.x

[52] McLaren DG, Ries ML, Xu G, Johnson SC (2012) A generalized form of context-dependent psychophysiological interactions (gPPI): a comparison to standard approaches. NeuroImage 61:1277–1286 22484411 10.1016/j.neuroimage.2012.03.068 PMC3376181

[53] Taylor KS, Seminowicz DA, Davis KD (2009) Two systems of resting state connectivity between the insula and cingulate cortex. Hum Brain Mapp 30:2731–2745 19072897 10.1002/hbm.20705 PMC6871122

[54] Ichesco E (2014) Altered resting state connectivity of the insular cortex in individuals with fibromyalgia. J Pain 15:815–826e1 24815079 10.1016/j.jpain.2014.04.007 PMC4127388

[55] Hayes AF (2018) Introduction to Mediation, Moderation, and Conditional Process Analysis: A Regression-Based Approach, 2nd edn. Guilford Press

